# Mycorrhizal trifoliate orange has greater root adaptation of morphology and phytohormones in response to drought stress

**DOI:** 10.1038/srep41134

**Published:** 2017-01-20

**Authors:** Ying-Ning Zou, Peng Wang, Chun-Yan Liu, Qiu-Dan Ni, De-Jian Zhang, Qiang-Sheng Wu

**Affiliations:** 1 College of Horticulture and Gardening, Yangtze University, Jingzhou, Hubei 434025, China; 2 Institute of Citrus Research, Zhejiang Academy of Agricultural Sciences, Taizhou, Zhejiang 318026, China; 3 Institute of Root Biology, Yangtze University, Jingzhou, Hubei 434025, China; 4 Department of Chemistry, Faculty of Science, University of Hradec Kralove, Hradec Kralove 50003, Czech Republic

## Abstract

Plant roots are the first parts of plants to face drought stress (DS), and thus root modification is important for plants to adapt to drought. We hypothesized that the roots of arbuscular mycorrhizal (AM) plants exhibit better adaptation in terms of morphology and phytohormones under DS. Trifoliate orange seedlings inoculated with *Diversispora versiformis* were subjected to well-watered (WW) and DS conditions for 6 weeks. AM seedlings exhibited better growth performance and significantly greater number of 1^st^, 2^nd^, and 3^rd^ order lateral roots, root length, area, average diameter, volume, tips, forks, and crossings than non-AM seedlings under both WW and DS conditions. AM fungal inoculation considerably increased root hair density under both WW and DS and root hair length under DS, while dramatically decreased root hair length under WW but there was no change in root hair diameter. AM plants had greater concentrations of indole-3-acetic acid, methyl jasmonate, nitric oxide, and calmodulin in roots, which were significantly correlated with changes in root morphology. These results support the hypothesis that AM plants show superior adaptation in root morphology under DS that is potentially associated with indole-3-acetic acid, methyl jasmonate, nitric oxide, and calmodulin levels.

Drought stress (DS), which is a key abiotic stress, is considered to be a major threat to crop growth and production worldwide^1^. Plant roots are usually the first plant parts to encounter DS. They are highly sensitive and responsive to DS conditions, which makes them intimately linked with the drought adaptation of the entire plant^2^. As a result, root growth and development are important for plants to survive soil DS. A deep and extensive root system enables plants to access moisture at water-absorbed zones in soils, and is thus considered as a vital strategy for drought adaptation^3^. In response to drought, root hairs often become shorter and more bulbous^4^. Recent advances in the understanding of DS effects have been achieved based on the evaluation of aboveground (shoot) traits, whereas little information is available about the effects of DS on root morphology and root hair development^1^.

The plant rhizosphere is inhabited by several soil microorganisms, including arbuscular mycorrhizal (AM) fungi. These fungi, belonging to the Glomeromycota, can colonize the roots of numerous terrestrial plants, forming mutualistic symbioses known as arbuscular mycorrhizas^5^. A number of studies have confirmed that AM fungi can enhance the drought tolerance of various plants species^6^,^7^,^8^. The potential mechanisms are: (1) direct water absorption by extraradical hyphae^9^,^10^, (2) increase in nutrient uptake, especially P^11^, (3) biochemical changes in osmotic adjustment and antioxidant defence systems^12^,^13^,^14^,^15^,^16^, (4) improvement of the soil structure by glomalin-related soil proteins of AM fungi^17^, and (5) changes in molecule levels, including aquaporins^7^,^18^,^19^, genes involved in oxidative stress^20^ and proline synthesis^21^, dehydrin proteins^22^, and genes involved in stomata development and ABA responses or encoding proteins involved in plant response pathways^23^,^24^. The underlying mechanisms of phytohormone responses and root adaptation need to be elucidated.

Plant roots have highly plastic traits that can be modulated by many factors, including AM fungi^25^. Earlier studies have showed that there are more branches in AM roots than in non-AM controls^26^,^27^. Root length, area, and volume were pronouncedly increased by *Funneliformis mosseae* in *Citrus tangerina* seedlings^28^. However, Atkinson *et al*.^29^ found no change in the root branching of *Trifolium repens* by *F. mosseae*. A decrease in the total root length and root diameter of rice plants was induced by *Rhizophagus intraradices*^30^. AM fungi also modulated greater root hair modification to improve aboveground growth^31^. These root modifications under mycorrhization could be linked to the improved uptake of nutrients^32^, sugar allocation to roots^33^, and polyamine metabolism^28^, independent of common symbiosis signalling^34^.

Phytohormones are associated with root formation and development, mycorrhizal formation, and response to DS^35^,^36^,^37^. AM symbiosis and methyl jasmonate (MeJA) prevented the inhibition of root hydraulic conductivity in *Phaseolus vulgaris* exposed to DS, probably through a reduction in root salicylic acid concentrations^38^. In addition, under unfavourable conditions, plants might stimulate increased strigolactone production to promote symbiosis to deal with the stress^8^. Mycorrhizal *P. vulgaris* plants had relatively higher indole-3-acetic acid (IAA) and abscisic acid (ABA) levels than non-mycorrhizal controls under well-watered (WW) and DS conditions^38^. Little is known about the response of phytohormones to mycorrhization under DS and the subsequent regulation of root morphology by these phytohormones.

Here, we hypothesize that roots of AM plants exhibit better adaptation of morphology in response to DS via modultion of phytohormone levels. The objective of the present study was to confirm the above hypothesis by examining the effects of AM fungi on lateral root formation, root morphology, and root hair development, as well as the relevant phytohormones and their analogues.

## Results

### Mycorrhizal status

Root colonization by *D. versiformis* ranged from 37.5% to 55.3% in AM seedlings, and DS treatment significantly (*P* < 0.05) decreased root colonization by 32.2% compared with WW (Table 1). Similarly, the soil hyphal length was significantly (*P* < 0.05) 42.3% higher under DS than under WW (Table 1).

### Plant growth performance

Compared with the WW treatment, the DS treatment significantly (*P* < 0.05) reduced plant height, stem diameter, number of leaves, and shoot and root biomass in both AM and non-AM seedlings. AM seedlings displayed significantly (*P* < 0.05) better growth traits than non-AM seedlings under WW and DS: 31% and 45% for plant height, 15% and 12% for stem diameter, 15% and 33% for the number of leaves, 42% and 52% for shoot biomass, and 37% and 32% for root biomass, respectively (Table 1).

### Leaf water potential (Ψ)

**Figure 1:**
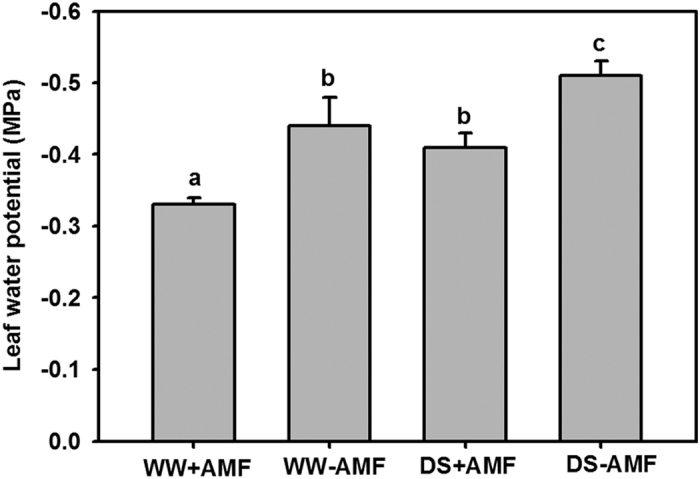
Leaf water potential of trifoliate orange (*P. trifoliata*) seedlings colonized by *D. versiformis* under well-watered and drought stress conditions. Data (means ± SD, *n* = 4) followed by different letters above the bars indicate significant differences (*P* < 0.05) between treatments. Abbreviations: +AMF: inoculation with *D. versiformis*. **−**AMF: inoculation without *D. versiformis*, DS: drought stress, WW: well-watered.

Compared with WW, DS significantly (*P* < 0.05) reduced leaf *Ψ* in trifoliate orange, irrespective of the AM fungi status (Fig. 1). Inoculation with AM fungi significantly (*P* < 0.05) increased leaf *Ψ* by 25% and 20% under WW and DS conditions, respectively.

### Number of lateral roots

**Figure 2:**
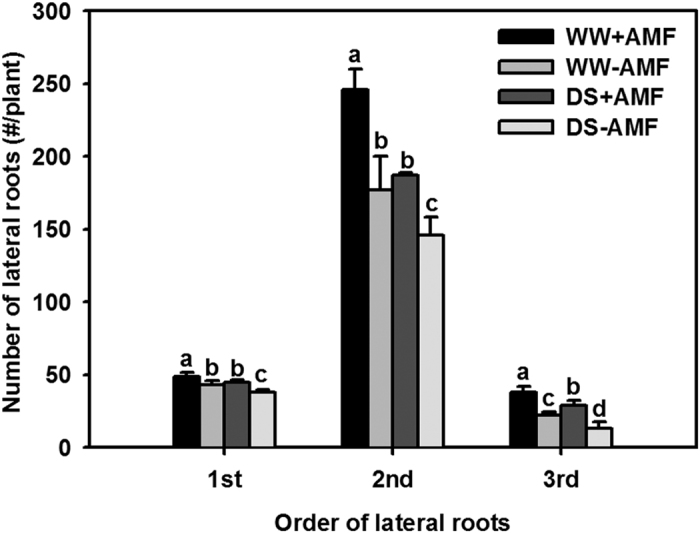
Number of 1^st^, 2^nd^ and 3^rd^-order lateral roots of trifoliate orange (*P. trifoliata*) seedlings colonized by *D. versiformis* under well-watered and drought stress conditions. Data (means ± SD, *n* = 4) followed by different letters above the bars indicate significant differences (*P* < 0.05) between treatments. Abbreviations: same as for Fig. 1.

In five-month-old trifoliate orange seedlings, the number of 2^nd^-order lateral roots was in the dominant position of the number of lateral roots (Fig. 2). DS treatment significantly (*P* < 0.05) decreased the number of 1^st^-, 2^nd^-, and 3^rd^-order lateral roots in both AM and non-AM seedlings, compared with the WW treatment. Compared with the non-mycorrhizal colonized controls, *D. versiformis* colonized seedlings showed a significant (*P* < 0.05) increase by 14%, 39%, and 73% in the number of 1^st^-, 2^nd^-, and 3^rd^-order lateral roots under WW, and by 18%, 28%, and 123% under DS, respectively.

### Root morphology

**Figure 3:**
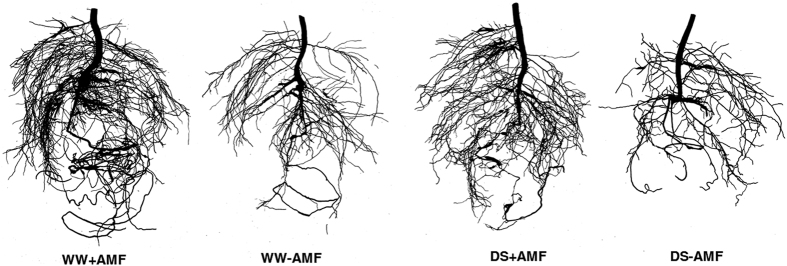
Root morphology status of trifoliate orange (*P. trifoliata*) seedlings colonized by *D. versiformis* under well-watered and drought stress conditions. Abbreviations: same as for Fig. 1.

Root morphology of five-month-old trifoliate orange seedlings was significantly reduced by the DS treatment, compared with the WW treatment, while it was increased by mycorrhizal inoculation under the same soil water status as compared with non-mycorrhizal treatment (Table 2; Fig. 3). AM seedlings represented significantly (*P* < 0.05) higher root length, projected area, surface area, average diameter, volume, tips, forks, and crossings than non-AM seedlings, regardless of whether the seedlings were subjected to DS: 13%, 8%, 9%, 5%, 66%, 323%, 91%, and 107% increase under WW, and 10%, 7%, 8%, 7%, 48%, 201%, 83%, and 79% under DS, respectively (Table 2).

### Root hair development

**Figure 4:**
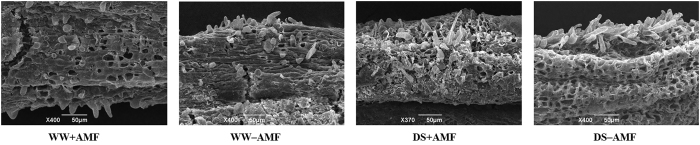
Root hair morphology status of trifoliate orange (*P. trifoliata*) seedlings colonized by *D. versiformis* under well-watered and drought stress conditions. Abbreviations: same as for Fig. 1.

**Figure 5:**
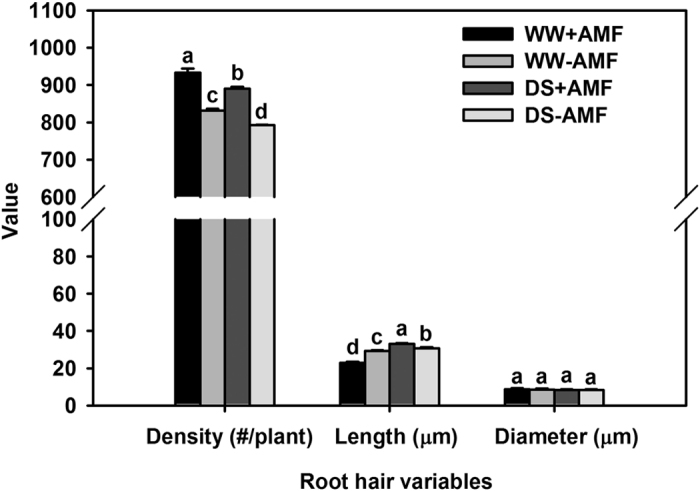
Root hair density, root hair length, and root hair diameter of trifoliate orange (*P. trifoliata*) seedlings colonized by *D. versiformis* under well-watered and drought stress conditions. Data (means ± SD, *n* = 4) followed by different letters above the bars indicate significant differences (*P* < 0.05) between treatments. Abbreviations: same as for Fig. 1.

Figure 4 showed the change in root hairs in response to the DS and mycorrhizal inoculation. DS treatment significantly reduced root hair density, while it notably (*P* < 0.05) increased root hair length and had no effect on root hair diameter, compared with WW (Fig. 5). Compared with the non-AM seedlings, root hair density in mycorrhizal colonized seedlings was significantly (*P* < 0.05) decreased by 12% under both WW and DS (Fig. 5). Root hair length was significantly (*P* < 0.05) 22% lower in AM seedlings than in non-AM seedlings under WW, whereas it was significantly 7% higher in AM seedlings than in non-AM seedlings under DS. Root hair length was not affected by AM fungal colonization, irrespective of the soil water status.

### Root endogenous phytohormone levels

**Figure 6:**
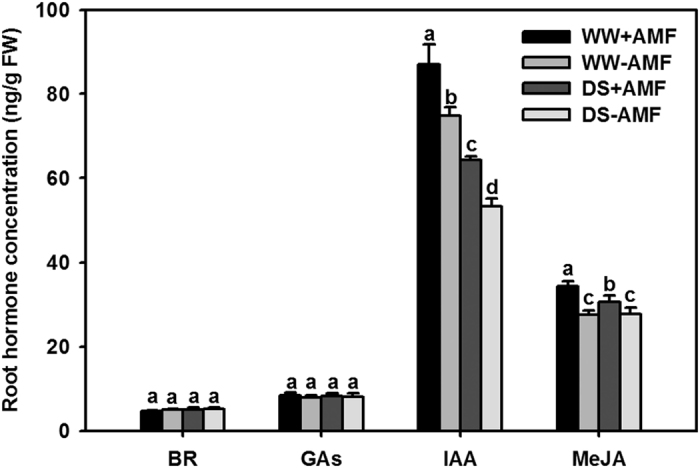
Root BR, GAs, IAA, and MeJA concentration of trifoliate orange (*P. trifoliata*) seedlings colonized by *D. versiformis* under well-watered and drought stress conditions. Data (means ± SD, *n* = 4) followed by different letters above the bars indicate significant differences (*P* < 0.05) between treatments. Abbreviations: same as for Fig. 1 and BR: brassinosteroid, GAs: gibberellins, IAA: indole-3-acetic acid, MeJA: methyl jasmonate.

Mycorrhizal inoculation and DS did not significantly (*P* < 0.05) alter the root brassinosteroid (BR) and gibberellins (GAs) concentrations (Fig. 6). The DS treatment induced lower accumulation of root IAA and MeJA than the WW treatment, irrespective of the AM status (Fig. 6). However, compared with non-mycorrhizal seedlings, root IAA concentration in mycorrhizal seedlings was increased, by 25% under WW and by 19% under DS. Root MeJA concentration was 24% and 11% higher under WW and DS in AM seedlings than in non-AM seedlings, respectively.

### Root calmodulin (CaM) and nitric oxide (NO) concentration

**Figure 7:**
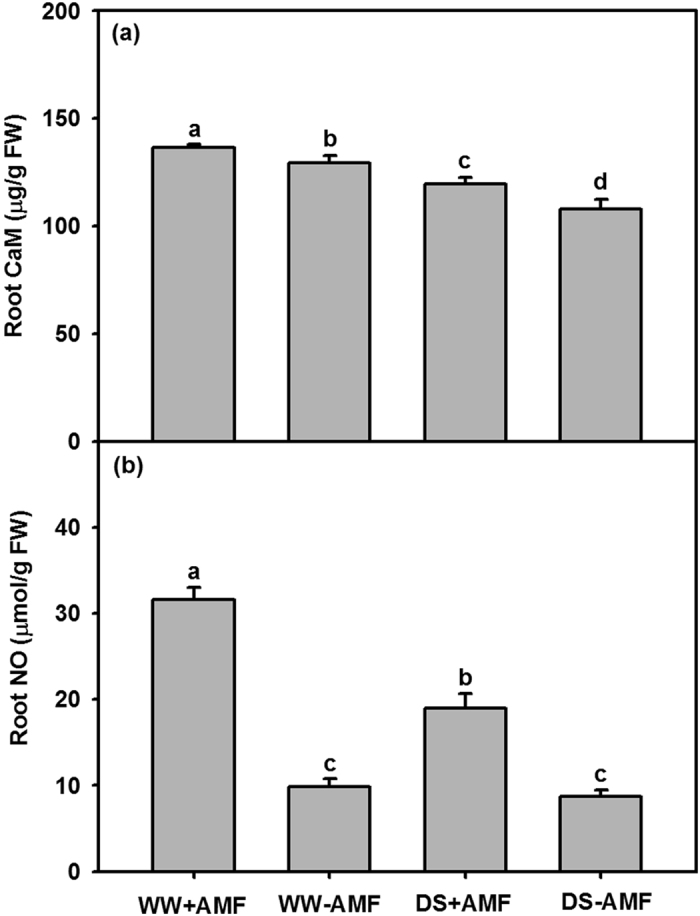
Root CaM and NO concentration of trifoliate orange (*P. trifoliata*) seedlings colonized by *D. versiformis* under well-watered and drought stress conditions. Data (means ± SD, *n* = 4) followed by different letters above the bars indicate significant differences (*P* < 0.05) between treatments. Abbreviations: same as for Fig. 1 and CaM: calmodulin; NO: nitric oxide.

The DS treatment significantly (*P* < 0.05) decreased the root calmodulin (CaM) and nitric oxide (NO) concentration in both AM and non-AM seedlings, as compared with the WW treatment (Fig. 7a,b). Compared with the non-AM fungal colonized controls, *D. versiformis*-colonized seedlings exhibited significantly (*P* < 0.05) higher root CaM concentration by 6% and 11% under WW and DS (Fig. 7a), and 219% and 117% higher root NO concentration under WW and DS (Fig. 7b), respectively.

### Correlation studies

Leaf *Ψ* and root IAA, MeJA, CaM, and NO, but not BR and GAs, showed a significantly (*P* < 0.01) positive correlation with the number of 1^st^-, 2^nd^-, and 3^rd^-order lateral roots, root morphological traits, and root hair density. In contrast, they showed a significantly (*P* < 0.01) negative correlation with root hair length and had no significant correlation with root hair diameter (Table 3).

## Discussion

In our study, inoculation with *D. versiformis* strongly stimulated the formation in 1^st^-, 2^nd^-, and 3^rd^-order lateral roots in trifoliate orange subjected to both WW and DS. This is in agreement with earlier studies reported in *Medicago truncatula* colonized by *Gigaspora margarita*^27^. Such mycorrhizal roots became progressively more branched than non-mycorrhizal controls^26^. This was confirmed by an increase in the number of tips, forks, and crossings in the present study. This shows that AM plants had a stronger adaptation in terms of lateral root formation than non-AM plants under DS. Mycorrhizal mycelium is mainly localized in the newly formed lateral roots^39^. Greater number of lateral roots in AM plants would provide better chances of colonization by AM fungi, as well as for absorbing water from the soil to the host plant, as shown in our study through a significant correlation between leaf *Ψ* and the number of lateral roots. A diffusible factor, ‘Myc factor’, from AM fungi, was found to stimulate lateral root formation^27^. Our study showed that root IAA, MeJA, CaM, and NO were involved in lateral root formation. As reported by Felten *et al*.^40^, mycorrhiza-stimulated lateral root induction is paralleled by an increase in the 1-naphthylphthalamic acid (NPA)-sensitive auxin response at the root apex and in provascular tissues, together with IAA-based auxin signalling.

The present study showed that exposure to DS for six weeks dramatically restricted root morphological development in trifoliate orange seedlings, irrespective of the AM status. This indicates that the volume of soils explored by the whole root systems was dramatically reduced by DS in soils, which is in agreement with earlier studies on rice^3^. However, the *D. versiformis* that colonized trifoliate orange seedlings caused an improvement in morphological traits (length, area, volume, diameter, tips, forks, and crossings), compared with the non-AM controls under both WW and DS, indicating a larger extensive distribution of roots in AM seedlings under DS, which can contribute to higher leaf *Ψ* than non-AM plants^41^. The improvement of the root system by mycorrhization was also found in split-root trifoliate orange seedlings colonized by *Acaulospora scrobiculata* and *F. mosseae*, grown in a two-chambered split-root system^42^. In general, root morphology, development, and physiology are closely connected with plant growth^41^. A better root morphology in AM plants would promote plant growth, as shown by the positive effect of AM fungi on plant growth performance in trifoliate orange under both WW and DS. As roots have the ability to grow toward the direction of high water availability in the soil^2^, a significantly positive correlation of root morphological traits with leaf *Ψ* suggests that roots of mycorrhizal plants can explore the soil better for water uptake under DS. As a result, AM plants possess a stronger capacity, owing to a better root morphology, to adapt to soil drought than non-AM plants.

Wu *et al*.^31^ showed that four AM fungal species (*Claroideoglomus etunicatum, D. versiformis, F. mosseae*, and *R. intraradices*) induced a significant increase in root hair density in trifoliate orange seedlings grown in sands. The present study further indicated a strong effect of AM fungal inoculation on the root hair density of trifoliate orange seedlings grown in soils under both WW and DS. Li *et al*.^43^ reported that AM fungi mainly enhanced plant drought tolerance by the improvement of P and leaf water status, but root hairs presumably contributed to the shoot P enhancement. The AM-mediated increase in root hair density does not depend on growth substrates and soil water status. On the other hand, AM fungal inoculation affected the root hair length with a significant decrease under WW and with a significant increase under DS, suggesting that the the effect of AM fungus on root hair length under WW can be reversed by DS, possibly due to the increase in nutrient requirements under poor nutrient-movement conditions. This reduction in root hair length under WW is consistent with the results of Sun and Tang^44^ in *Sorghum bicolor* plants inoculated with *F. mosseae* and *R. intraradices*. The increase in AM-induced root hair length under DS is in agreement with the results of Wu *et al*.^31^. We also concluded that higher concentrations of IAA, MeJA, NO, and CaM in roots under mycorrhization stimulate AM fungal colonization under WW and also improve root morphology under DS, indicating different functionings of these phytohormones in mycorrhizal plants exposed to water treatments.

Earlier studies confirmed a negative effect of GAs on root mycorrhizal colonization^45^,^46^ and a limited role of BR in determining AM development^31^. In this study, both AM fungal inoculation and water treatment produced no changes in the root BR and GAs concentration of trifoliate orange seedlings, suggesting that BR and GAs are not key factors involved in mycorrhizal development.

The root IAA level, in this study, was significantly decreased by DS while it was increased by AM fungal colonization, regardless of the soil water status. The mycorrhiza-induced root IAA increase might be due to small amounts of IAA that could be released by spores of AM fungi^47^. IAA, the major endogenous auxin in plants, is required for root development and root hair formation^48^. Moreover, auxins participate in the induction of cellular rhizogenic competence, root apical meristem differentiation, and development of the root cap and vasculature^49^. A mycorrhiza-induced IAA increment in roots would improve root morphology and lead to root hair modification. Therefore, it is reasonable that root IAA was significantly and positively correlated with root morphological traits and root hair density. Root IAA was negatively correlated with root hair length, whereas root hair length was significantly decreased by AM fungi under WW and increased under DS, suggesting that AM-induced IAA changes are dependent on soil water status and the integrated effects of several phytohormones, besides IAA. In addition, auxin accumulation could be involved in plant responses to DS via the activation of signalling pathways and induction of auxin-responsive genes^50^. The increase in the root IAA by mycorrhization has the potential capacity to enhance drought tolerance of the host plant.

In addition to IAA, NO participates in the induction of root tip elongation and the formation of lateral roots^51^. It is also a critical molecule for root hair formation through the auxin-signalling cascade^52^. In this study, in spite of the negative effect of DS on root NO levels, root NO concentrations were significantly increased in response to AM fungal inoculation under both WW and DS. This is consistent with the results of Calcagno *et al*.^53^ in *Medicago truncatula* colonized by *Gigaspora margarita* and Espinosa *et al*.^54^ in olive seedlings inoculated with *R. irregularis*. It has been established that NO is involved in root mycorrhizal colonization. Furthermore, root NO was significantly and positively correlated with the number of lateral roots, root morphological traits, and root hair density. This implies that mycorrhizal colonization heavily stimulated root NO production, which might play a role in the formation of lateral roots and root hairs. The NO-mediated effect on roots is under the control of auxins^52^. Further works are needed to clarify the mycorrhiza-induced root improvement by NO-mediated auxin signalling^55^. In addition, induction of root NO accumulation under mycorrhization might be a part of the mechanism producing a local response to enhance drought tolerance in plants^51^.

Methyl jasmonate (MeJA) has been identified as a vital cellular regulator to mediate developmental processes (including root hair production), proper arbuscule formation^44^, and defence responses against stresses (e.g., drought)^56^. Our study indicated that the root MeJA concentrations in trifoliate orange seedlings were pronouncedly increased by the AM fungal inoculation, irrespective of the soil water status. This is in agreement with previous studies on barley, soybean, and trifoliate orange plants under mycorrhization^31^,^57^,^58^. The root MeJA concentration was significantly and positively correlated with root hair density, but was negatively correlated with root hair length, which is in agreement with our previous work on trifoliate orange^31^. The increase in root MeJA concentration upon mycorrhizal inoculation might be associated with improving drought tolerance of the host plant, as previously mentioned by Anjum *et al*.^59^.

An earlier study had reported an increase in the intracellular CaM in soybean after being colonized by *R. intraradices*^60^. The present study also indicated that inoculation with *D. versiformis* induced the accumulation of root CaM under both WW and DS. Similar results were found in trifoliate orange seedlings colonized by *F. mosseae* under both WW and DS^16^. It seems that CaM is involved in the process of root mycorrhizal colonization. Lévy *et al*.^61^ further confirmed that Ca^2+^ spiking and CaM-dependent protein kinases are necessary for mycorrhizal infection. Calmodulin (CaM), a Ca^2+^ receptor, can bind with Ca^2+^ as the Ca^2+^/CaM messenger system to activate downstream target proteins, thereby regulating the generation of reactive oxygen species and modulating transcription factors to maintain homeostasis between different cellular processes^62^. In the study of Huang *et al*.^16^, mycorrhiza-induced CaM mediated the antioxidant enzyme defence system to enhance drought tolerance in plants. Higher root CaM levels in AM plants have the capacity to enhance drought tolerance of the host plant. CaM is a primary decoder of Ca^2+^ signals^63^, while the Ca^2+^ gradient and Ca^2+^ influxes induce root hair formation and growth^64^. Root CaM was significantly and positively correlated with root hair density while negatively correlated with root hair length.

In short, our results supported the preceding hypothesis that AM plants had better root adaptation of morphology in response to drought stress, which is potentially associated with AM-induced changes in IAA, MeJA, NO, and CaM. It is therefore suggested that in citriculture, either stimulating the formation and development of AMs or inoculating native AM fungi into citrus orchards, would be vital for the enhancement of drought tolerance and root morphology in citrus trees.

## Methods

### Plant culture

Seeds of trifoliate orange were sterilized by 70% of ethanol solution for 10 min, rinsed 4 times with distilled water, and pre-germinated in autoclaved (121 °C, 0.11 Mpa, 2 h) river sand under the condition of 27/20 °C day/night temperature, 740 μmol/m^2^/s photon flux density, and 80% relative humidity. After 4 weeks, three five-leaf-old seedlings were transplanted into a plastic pot (11.5 cm upper diameter × 8.5 cm bottom diameter × 14 cm height) and supplied with 1.8 kg of autoclaved (0.11 Mpa, 121 °C, 2 h) soil + vermiculite mixture (2:1, v/v) on March 28, 2014. The soil, which belongs to the Xanthi-udic Ferralsol soil (FAO system), was collected from a citrus orchard on the Yangtze University campus (30°36′N, 112°14′E, 36 m above sea level). The soil has the characteristics of pH 6.0, 12.1 mg/kg KMnO_4_-N, 15.7 mg/kg Bray-P, and 22.3 mg/kg neutral NH_4_OAc-K. Half of the seedlings received the AM fungal inoculated treatment. A 2200-spore dosage of an AM fungus, *Diversispora versiformis* (P. Karst.) Oehl, G. A. Silva & Sieverd, was applied into the surroundings of the plant roots. The AM fungus was supplied by the Bank of Glomeromycota in China (BGC). The AM fungus was isolated from the rhizosphere of *Astragalus adsurgens* in Ejin Horo Banner, Inner Mongolia Autonomous Region, China. The spores of the AM fungus were propagated by white clover in a mixture of sand and soil (1:1, v/v) for 12 weeks. For the non-AM fungal treatment, seedlings were supplied the same amount of autoclaved AM fungal inocula plus 2 mL filtrate (25 μm filter) of mycorrhizal inoculum to minimize differences in other microbial communities. AM and non-AM seedlings were placed in a green house on the Yangtze University campus, where the photosynthetic photon flux density was 880 μmol/m^2^/s, day/night temperature 28/21 °C, and relative humidity 85%. During the experiment, a 30 mL Hoagland solution per pot was used to replace 30 mL distilled water weekly.

### Water treatments

After AM fungal inoculation, the seedlings were gravimetrically maintained at 75% of maximum water holding capacity (soil WW status) in the growth substrate for 15 weeks. Subsequently, half of AM and non-AM seedlings were changed to 50% of maximum water holding capacity (soil DS status) in the growth substrate for DS for 6 weeks. The other seedlings were still kept in soil WW status for 6 weeks. After 21 weeks of water treatments, seedlings were harvested.

### Experimental design

The experiment was composed of AM fungal inoculations (with or without *D. versiformis*) and water treatments (WW and DS) with a completely randomized arrangement, for a total of four treatments. Each treatment was replicated four times, for a total of 16 pots.

### Determinations of plant growth performance

Before harvest, plant height, stem diameter, and number of leaves per plant were recorded. At harvest, the seedlings were divided into shoots and roots, whose biomass was determined.

### Determination of mycorrhizas

One cm long root segments were cleared with 10% (w/v) KOH in 95 °C for 1.5 h and stained with 0.05% (w/v) trypan blue in lactoglycerol for 5 min^65^. Root AM fungal colonization was calculated as the percentage of AM fungal colonized root lengths against total root lengths.

### Determination of root morphology

After washing with tap water, the intact root system of each seedling from each pot was placed in a Regent’s water-proof plexiglass tray without root overlap and then scanned by an EPSON Flatbed Scanner, Epson Perfection V700 (Seiko Epson Corp, Japan). The root images were analyzed by the WinRHIZO 2007d (Regent Instruments Incorporated, Quebec, Canada) for root morphological variables, including total length, projected area, surface area, average diameter, and volume. Lateral roots grew on the taproot were gradually defined as 1^st^, 2^nd^, and 3^rd^ order, and the number of different order lateral roots was counted through placing experimental tables.

### Determination of root-hair morphology

Root hairs were observed as per the protocol outlined by Wu *et al*.^31^. At harvest, the root-hair zone was quickly collected and fixed with 2.5% glutaraldehyde with 0.1 mol/L sodium cacodylate buffer (pH 7.4), dehydrated step by step by alcohol with increasing concentrations, and dried using critical-point drying (CPD). A scanning electron microscope (SEM, model JSM-6390LV, JEOL Co., Japan) was used to acquire the photo of root hairs. The root hair photo was analyzed by the Image J software (National Institutes of Health, Maryland, USA) for density, length, and diameter of root hairs.

### Determination of leaf water potential (Ψ)

The leaf water potential (leaf *Ψ*) was measured by a PSΨPRO Water Potential System with a leaf hygrometer (L-51A-SF, WESCOR).

### Determination of root endogenous hormones

Extraction of IAA, GAs, BR, and MeJA was done by the protocol described by Wu *et al*.^31^ and determined by the enzyme-linked immunosorbent assay (ELISA). These ELISAs were provided by the Engineering Research Center of Plant Growth Regulator, China Agricultural University.

Root NO and CaM levels were determined by the ELISA assay (CaM: YAD-001, Beijing Dingguochangsheng Biotechnology Co., Ltd, Beijing, China; NO: A012, Nanjing Jiancheng Bioengineering Institute, Nanjing, China).

### Statistical analysis

Data (means ± SD, *n* = 4) were statistically analyzed by two-factor variance (ANOVA) in SAS software (SAS Institute Inc., Cary, NC, USA). Data regarding root AM fungal colonization were arcsine transformed prior to ANOVA. The Duncan’s multiple range tests at *P* < 0.05 were utilized to compare significant differences between treatments.

## Additional Information

**How to cite this article:** Zou, Y.-N. *et al*. Mycorrhizal trifoliate orange has greater root adaptation of morphology and phytohormones in response to drought stress. *Sci. Rep.* **7**, 41134; doi: 10.1038/srep41134 (2017).

**Publisher's note:** Springer Nature remains neutral with regard to jurisdictional claims in published maps and institutional affiliations.

## Figures and Tables

**Table 1:** Mycorrhizal status and plant growth performance of *D. versiformis* colonized trifoliate orange seedlings in response to different treatments.

Treatments	Mycorrhizal status		Plant growth performance				
	Root colonization (%)	Soil hyphal length (m/g DW soil)	Plant height (cm)	Stem diameter (mm)	Leaf number (#/plant)	Shoot biomass (g FW/plant)	Root biomass (g FW/plant)
WW + AMF	55.3 ± 8.8a	0.52 ± 0.06a	29.1 ± 2.6a	4.16 ± 0.11a	22.8 ± 1.2a	2.47 ± 0.21a	2.61 ± 0.13a
WW–AMF	0c	0c	20.1 ± 2.9c	3.52 ± 0.07c	19.4 ± 2.2b	1.43 ± 0.13c	1.65 ± 0.16c
DS + AMF	37.5 ± 7.6b	0.30 ± 0.04b	25.8 ± 1.2b	3.80 ± 0.15b	21.2 ± 1.5ab	2.13 ± 0.12b	2.00 ± 0.10b
DS–AMF	0c	0c	14.1 ± 1.0d	3.34 ± 0.11d	14.3 ± 1.5c	1.02 ± 0.08d	1.36 ± 0.08d

Data (means ± SD, *n* = 4) followed by different letters indicate significant differences among treatments after using the Duncan’s multiple range test (*P* < 0.05). Abbreviations: +AMF: inoculation with *D. versiformis*, –AMF: inoculation without *D. versiformis, D*S: drought stress, WW: well-watered.

**Table 2:** Root morphological traits of *D. versiformis* colonized trifoliate orange seedlings in response to different treatments.

Treatments	Length (cm)	Projected area (cm^2^)	Surface area (cm^2^)	Average diameter (mm)	Volume (cm^3^)	Tips (#/plant)	Forks (#/plant)	Crossings (#/plant)
WW + AMF	240 ± 15a	13.7 ± 0.3a	17.6 ± 0.4a	0.565 ± 0.012a	1.73 ± 0.06a	2119 ± 220a	2562 ± 238a	765 ± 58a
WW − AMF	213 ± 5b	12.7 ± 0.1b	16.2 ± 0.2c	0.537 ± 0.013b	1.04 ± 0.11c	501 ± 84c	1338 ± 123c	370 ± 24b
DS + AMF	216 ± 7b	12.9 ± 0.2b	16.7 ± 0.2b	0.548 ± 0.005b	1.29 ± 0.11b	889 ± 46b	2006 ± 90b	451 ± 63b
DS − AMF	196 ± 8c	12.1 ± 0.4c	15.5 ± 0.2d	0.514 ± 0.004c	0.87 ± 0.03d	295 ± 103d	1097 ± 71d	252 ± 30c

Data (means ± SD, *n* = 4) followed by different letters indicate significant differences among treatments after using the Duncan’s multiple range test (*P* < 0.05). Abbreviations: same as Table 1.

**Table 3:** Pearson’s correlations (*r*) between root morphological traits and leaf *Ψ* or root chemical variables (*n* = 16).

Variables	Root BR	Root GAs	Root IAA	Root MeJA	Root CaM	Root NO	Leaf *Ψ*
Number of lateral roots							
1^st^	−0.32	0.39	0.79**	0.66**	0.82**	0.80**	0.83**
2^nd^	−0.41	0.35	0.82**	0.86**	0.79**	0.90**	0.86**
3^rd^	−0.40	0.23	0.93**	0.61*	0.93**	0.71**	0.79**
Root morphology							
Length	−0.53*	0.20	0.88**	0.72**	0.84**	0.79**	0.83**
Projected area	−0.37	0.23	0.91**	0.73**	0.87**	0.76**	0.85**
Surface area	−0.46	0.41	0.84**	0.81**	0.80**	0.90**	0.95**
Average diameter	−0.29	0.35	0.81**	0.74**	0.72**	0.82**	0.88**
Volume	−0.28	0.30	0.80**	0.90**	0.72**	0.94**	0.89**
Tips	−0.38	0.21	0.92**	0.78**	0.85**	0.84**	0.84**
Forks	−0.36	0.28	0.71**	0.90**	0.66**	0.95**	0.85**
Crossings	−0.38	0.28	0.86**	0.88**	0.80**	0.94**	0.88**
Root hair morphology							
Density	−0.38	0.34	0.74**	0.84**	0.72**	0.94**	0.91**
Length	0.33	−0.19	−0.81**	−0.64**	−0.74**	−0.70**	−0.66**
Diameter	−0.19	−0.02	0.20	0.10	0.28	0.18	0.16

^**^P < 0.01. Abbreviations: BR: brassinosteroid, CaM: calmodulin, GAs: gibberellins, IAA: indole-3-acetic acid, NO: nitric oxide, MeJA: methyl jasmonate, *Ψ*: water potential.
